# Overtreatment in nonmalignant lesions detected in a colorectal cancer screening program: a retrospective cohort study

**DOI:** 10.1186/s12885-021-08606-w

**Published:** 2021-07-29

**Authors:** Joaquín Cubiella, Antía González, Raquel Almazán, Elena Rodríguez-Camacho, Raquel Zubizarreta, Isabel Peña-Rey Lorenzo

**Affiliations:** 1 Department of Gastroenterology, Hospital Universitario de Ourense, Rúa Ramón Puga 52-56, 32003 Ourense, Spain; 2 Instituto de Investigación Sanitaria Galicia Sur, Ourense, Spain; 3 Centro de Investigación Biomédica en Red Enfermedades Hepáticas y Digestivas, Ourense, Spain; 4 Department of Preventive Medicine, Hospital Universitario de Ourense, Ourense, Spain; 5 Dirección Xeral de Saúde Pública, Conselleria de Sanidade, Santiago de Compostela, Spain

**Keywords:** Adenoma detection rate, Colorectal cancer, Overtreatment, Screening, Surgical resection

## Abstract

**Background:**

Although colorectal cancer (CRC) screening programs reduce CRC incidence and mortality, they are associated with risks in healthy subjects. However, the risk of overtreatment and overdiagnosis has not been determined yet. The aim of this study was to report the surgery rates in patients with nonmalignant lesions detected within the first round of a fecal immunochemical test (FIT) based CRC screening program and the factors associated with it.

**Methods:**

We included in this analysis all patients with nonmalignant lesions detected between May 2013 and June 2019 in the Galician (Spain) CRC screening program. We calculated surgery rate according to demographic variables, the risk classification according to the colonoscopy findings (European guidelines for quality assurance), the endoscopist’s adenoma detection rate (ADR) classified into quartiles and the hospital’s complexity level. We determined which variables were independently associated with surgery rate and expressed the association as Odds Ratio and its 95% confidence interval (CI).

**Results:**

We included 15,707 patients in the analysis with high (19.9%), intermediate (26.9%) low risk (23.3%) adenomas and normal colonoscopy (29.9%) detected in the analyzed period. Colorectal surgery was performed in 162 patients (1.03, 95% CI 0.87–1.19), due to colonoscopy complications (0.02, 95% CI 0.00–0.05) and resection of colorectal benign lesions (1.00, 95% CI 0.85–1.16). Median hospital stay was 6 days with 17.3% patients developing minor complications, 7.4% major complications and one death. After discharge, complications developed in 18.4% patients. In benign lesions, an endoscopic resection was performed in 25.4% and a residual premalignant lesion was detected in 89.9%. The variables independently associated with surgery in the multivariable analysis were age (≥60 years = 1.57, 95% CI 1.11–2.23), sex (female = 2.10, 95% CI 1.52–2.91), the European guidelines classification (high risk = 67.94, 95% CI 24.87–185.59; intermediate risk = 5.63, 95% CI 1.89–16.80; low risk = 1.43; 95% CI 0.36–5.75), the endoscopist’s ADR (Q4 = 0.44, 95% CI 0.28–0.68; Q3 = 0.44, 95% CI 0.27–0.71; Q2 = 0.71, 95% CI 0.44–1.14) and the hospital (tertiary = 0.54, 95% CI 0.38–0.79).

**Conclusions:**

In a CRC screening program, the surgery rate and the associated complications in patients with nonmalignant lesions are low, and related to age, sex, endoscopic findings, endoscopist’s ADR and the hospital’s complexity.

**Supplementary Information:**

The online version contains supplementary material available at 10.1186/s12885-021-08606-w.

## Background

Colorectal cancer (CRC) is the second most frequent cancer worldwide with almost two million incident cases and one million related deaths in 2020 [1]. In order to reduce the disease burden, population-based CRC screening programs have been established in the Western world. This strategy has demonstrated their efficacy to reduce CRC mortality and incidence in randomized controlled trials [2]. Furthermore, CRC screening programs have demonstrated their efficiency in reducing both CRC mortality and incidence [3, 4].

Screening programs are directed to asymptomatic subjects. A key point o is the minimization of risks. The benefit gained by individuals should outweigh any harm [5]. Although complications related to the diagnostic tests are well established in CRC screening [2, 6], there is no such certainty regarding overdiagnosis and overtreatment. Overdiagnosis is defined as the diagnosis of a medical condition or disease that would not cause symptoms or death during a patient’s lifetime. In the case of CRC screening, treatment of overdiagnosed CRC and polyps should be called overtreatment [7].

Endoscopic resection of colorectal polyps is the key to reduce CRC incidence and mortality [8]. Although side effects are limited, mainly postpolypectomy syndrome, rectal bleeding and perforation, they account for most colonoscopy-related injury during CRC screening [2]. Endoscopic resection removes up to 90% of advanced complex polyps [9]. However, the introduction of CRC screening programs has increased the number of colectomies due to benign polyps. In the US, up to 25% of colectomies were performed for non-malignant polyps [10]. Related mortality and morbidity attains 0.8 and 25.3%, respectively [11]. However, there is little information regarding the incidence of surgery and associated risks in subjects with benign lesions detected in a CRC screening program [12] Thus, we decided to perform a retrospective cohort analysis in the first round of the Galician (northwestern Spain) CRC screening program to determine the surgery rate in patients with non-malignant lesions detected on colonoscopy, the surgery-related complications, the motivation for surgery and finally, the factors independently associated with it.

## Methods

### Study design

We designed a retrospective cohort multicenter study using the Galician (Northwestern Spain) CRC screening program database to identify patients. We included in this analysis all patients that underwent at least one colonoscopy in the first round of the CRC screening program from its implementation (May 2013) until July 2019. We excluded patients with an invasive CRC as the final diagnosis.

### Description of the Galician CRC screening program

Galician CRC mass screening and its implementation have been described elsewhere [13]. The CRC screening program includes the central coordination and management of patient follow-up after polyp resection depending on their risk according to EU guidelines for quality assurance on CRC screening recommendations [14]. The Coordination Unit personnel introduces the data obtained from the different sources in the screening program information system regarding CRC stage according to the AJCC classification [15], the final classification of patients with a positive result [14] as well as several quality endoscopist indicators according to Spanish guidelines on quality in screening colonoscopy [16].

### Baseline data

From each patient, we collected the information available in the screening program database: sex, age, fecal hemoglobin concentration, performance status, associated medical illnesses graded according to the American Society of Anesthesiologists’ Physical Status Classification (ASA grade), number of baseline colonoscopies (colonoscopies performed after the positive FIT result), number of polyps, adenomas and size of the largest adenoma. Patients were classified as high risk (≥ 20 mm or ≥ 5 adenomas), intermediate risk (3 to 4 adenomas, 1 ≥ 10 mm and < 20 mm, with a villous component or high grade dysplasia), low risk (1–2 tubular adenomas < 10 mm in size) and no adenomas according to the European guidelines for quality assurance in CRC screening [17]. Data regarding the center and the endoscopist that performed the first complete colonoscopy were collected. The adenoma detection rate (ADR) and number of colonoscopies performed during the first round were calculated for each endoscopist in the first round. Endoscopists were classified into quartiles according to their ADR and number of colonoscopies performed. Finally, hospitals were classified according to their complexity level (tertiary versus secondary).

### Surgery

We identified all the patients that required surgery after colonoscopy using the Spanish Health System′s Hospital Discharge Records Database (CMBD in Spanish) and the CRC screening program database. The CMBD includes information on hospital discharges using a list of clinical codes to establish the diagnosis that justified the admission The CMBD database receives notifications from approximately 98% of Spanish public hospitals [18]. Mandatory health insurance covers an estimated 99.5% of the Spanish population, although subjects not covered by health insurance can still receive treatment in public hospitals. All subjects included in the Galician CRC screening programme are attended in the Galician Public health System. The International classification of diseases (ICD) codes used to identify colorectal surgeries were: ICD-9-MC 48.6 over the period 2013–2015 and ICD-10-ES ODT(C,E-N,P)(0,4) ZZ; ODB(C,E-N,P)(0,4) ZZ and ODBP7ZZ over the period 2016–2019. We subsequently searched manually the clinical records of identified patients to confirm that colorectal surgery was related to the screening colonoscopy. Moreover, we retrieved the following data: reason for surgery, type of surgery, length of hospital stay and complications either during hospitalization or after discharge (first year). We searched the clinical information in IANUS, the Galician electronic health record system that covers both all the Galician hospitals and the primary healthcare centers. Inhospital complications were classified according to the Clavien-Dindo classification [19]. We classified surgery complications as minor if they were grade I-II and major if they were grade III-V. If surgery was due to resection of colorectal lesions, we collected data regarding size, morphology according to the Paris classification [20], location, endoscopic resection and histologic findings in the endoscopic and surgical specimen. Based on endoscopic reports we calculated the Size, Morphology, Site and Access (SMSA) score and we classified lesions accordingly [21].

### Analysis

First, we described the characteristics of the subjects included. We reported continuous and categorical variables as median and interquartile range (IQR), and total number and percentage, respectively. Thereafter, we calculated the surgery rate according to the different variables assessed. We performed a bivariate analysis using the Chi-square test for categorical variables and the Student’s *t* test for continuous variables to determine those related to surgery. Finally, we included statistically significant or clinically relevant variables in a multivariable analysis using logistic regression (forward conditional) to determine which variables were independently related to surgery. We performed a secondary analysis after excluding transanal surgery to determine the colectomy rate, related complications and independently associated factors. Associations were expressed as Odds Ratio (OR) with a 95% confidence interval (CI). Statistical analyses were performed with IBM SPSS Statistics for Windows, Version 22.0. Armonk, NY, USA: IBM Corp.

### Ethics issues

The local Institutional Review Board assessed and approved the study (code 2018/593). As long as the study was based on database operation, no informed consent was required. The information was accessed according to prevailing European and Spanish legislation.

## Results

### Description of the sample

**Fig. 1:**
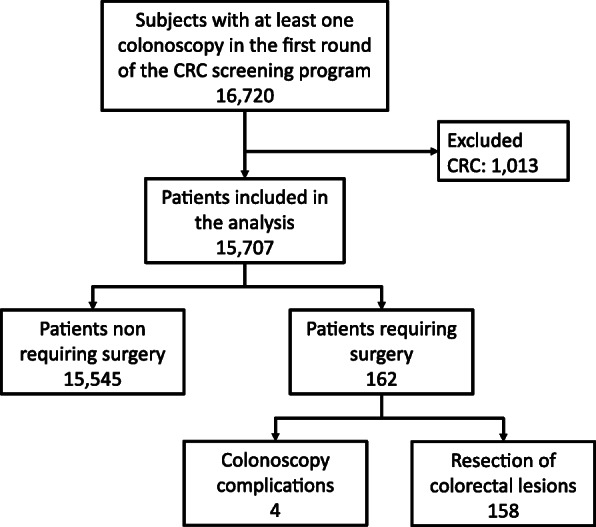
Flowchart of patients included in the analysis

Between May 2013 and June 2019, a total of 16,720 subjects underwent at least one colonoscopy during the first round of the Galician CRC screening program. We excluded 1013 subjects with CRC as the final diagnosis from this analysis. Therefore, we included in the analysis 15,707 patients without a CRC in the first round. After linking this data with the CMBD database, we identified 352 patients with any of the codes related to colorectal surgery. After verifying the clinical records, we confirmed that 162 underwent colorectal surgery related to participation in the screening program, four due to colonoscopy-related complications and 158 due to resection of colorectal lesions (Fig. 1 and Table 1). The surgery rate was as follows: global: 1.03% (95% CI 0.87–1.19), due to colonoscopy complications: 0.02% (95% CI 0.00–0.05) due to resection of colorectal lesions: 1.00% (95% CI 0.85–1.16). In the seven hospitals taking part in the CRC screening program, the surgery rate ranged across participating hospitals between 0.27% (95% CI 0.05–0.50) and 1.89% (95% CI 1.36–2.43). After excluding transanal surgeries (31) the colectomy rate was 0.83% (95% CI 0.69–0.98). The colectomy rate again ranged between 0.23% (95% CI 0.03–0.43) and 1.62% (95% CI 1.06–2.17). In Table 1, we outline the characteristics of the sample as well as the surgery rate according to dependent variables and in Supplementary Table 1 we show the same results referred to colectomies.

**Table 1:** Baseline characteristics and surgery rate according to dependent variables

		Patients not requiring surgery (*n* = 15,545)	Surgery (*n* = 162)	Surgery rate % (95% CI)	*Significance*^a^
Sex (*n* = 15,707)	• Female	6708 (43.2%)	72 (44.4%)	1.06 (0.82–1.31)	0.7
	• Male	8837 (56.8%)	90 (55.6%)	1.01 (0.80–1.22)	
Age (*n* = 15,707)	• < 60 years	6855 (44.1%)	48 (29.6%)	0.69 (0.50–0.89)	< 0.001
	• ≥60 years	8690 (55.9%)	114 (70.4%)	1.29 (1.06–1.53)	
PS (*n* = 15, 383)	• 0	12,388 (81.3%)	132 (83.0%)	1.05 (0.87–1.23)	0.8
	• 1	2836 (18.6%)	27 (17.0%)	0.94 (0.59–1.29)	
ASA (*n* = 15,383)	• I	8876 (58.2%)	96 (60.3%)	1.07 (0.86–1.28)	0.9
	• II	5391 (35.4%)	53 (33.3%)	0.97(0.71–1.23)	
	• III	957 (6.3%)	10 (6.3%)	1.03 (0.40–1.67)	
Fecal Hb (*n* = 15,707)	• < 100 μg/g	10,388 (66.8%)	79 (48.8%)	0.75 (0.59–0.92)	< 0.001
	• 100–200 μg/g	2935 (18.9%)	41 (25.3%)	1.42 (0.99–1.86)	
	• > 200 μg/g	2222 (14.3%)	42 (25.9%)	1.85 (1.30–2.41)	
N.colonoscopies (*n* = 15,707)	• One	13,557 (87.2%)	122 (75.3%)	0.89 (0.73–1.05)	< 0.001
	• At least two	1988 (12.8%)	40 (24.7%)	1.97 (1.37–2.58)	
Number of polyps (*n* = 15,707)		2 (IQR 1–4)	3 (IQR 1–5)		< 0.001
Number of adenomas (*n* = 15,707)		1 (IQR 0–3)	2 (IQR 1–4)		< 0.001
Adenoma size (mm) (*n* = 15,707)		5 (IQR 0–12)	19 (IQR 8–31.3)		< 0.001
European guidelines classification (*n* = 15,707)	• No adenoma	4650 (29.9%)	4 (2.5%)	0.08 (0.00–0.17)	< 0.001
	• Low risk	3626 (23.3%)	4 (2.5%)	0.11 (0.00–0.22)	
	• Intermediate risk	4179 (26.9%)	17 (10.5%)	0.40 (0.21–0.59)	
	• High risk	3090 (19.9%)	137 (84.6%)	4.24 (3.55–4.94)	
Endoscopist’s ADR (*n* = 15,459)	• Q1 (≤60%)	2653 (17.3%)	42 (26.1%)	1.56 (1.09–2.03)	0.02
	• Q2 (60–65.3%)	3989 (26.1%)	40 (24.8%)	0.99 (0.67–1.30)	
	• Q3 (65.3–70.8%)	4181 (27.3%)	34 (21.1%)	0.81 (0.54–1.08)	
	• Q4 (> 70.8%)	4474 (29.2%)	45 (28.0%)	0.99 (0.71–1.28)	
Endoscopist’s number of colonoscopies (*n* = 15,459)	• Q1 (≤57)	198 (1.3%)	2 (1.2%)	1.00 (−0.38–2.38)	0.03
	• Q2 (58–278)	1647 (10.8%)	18 (11.2%)	1.08 (0.58–1.58)	
	• Q3 (279–507)	5340 (34.9%)	39 (24.2%)	0.72 (0.50–0.95)	
	• Q4 (> 507)	8113 (53.0%)	102 (63.4%)	1.24 (1.00–1.48)	
Complexity of hospital (*n* = 15,707)	• Third level	7150 (46.0%)	52 (32.1%)	0.72 (0.53–0.92)	< 0.001
	• Second level	8395 (54.0%)	110 (67.9%)	1.29 (1.05–1.53)	

*ADR* Adenoma detection rate, *ASA* American Society of Anesthesiologists’ Physical Status, *CI* Confidence interval, *Hb* Hemoglobin, *IQR* Interquartile range, *PS* Performance status

^a^Significance in the univariant analysis using the Chi-square test for qualitative variables and the Student’s t test for cuantitative variables

Seventy-one endoscopists from seven hospitals took part in the first round of the CRC screening program. The median number of colonoscopies performed was 278 (IQR 56–507) and the median ADR was 65.3% (IQR 60.0–70.08%). We classified endoscopists into quartiles according to number of colonoscopies performed and ADR. Finally, hospitals were classified into tertiary (three) and secondary (four).

### Type of surgery and complications

As we show in Table 2, the main surgical approach was laparoscopy (57.4%). The most common surgeries performed were right-sided interventions (51.3%) and transanal resections (19.1%). The median length of hospitalization was 6 days with minor and major complications in *n* = 28 (17.3%) and *n* = 12 (7.4%) patients, respectively. Only one patient died due to surgery. After discharge, complications were detected in *n* = 29 (18.1%) patients, mainly due to intestinal subocclusion (5), rectal bleeding (3), abdominal wall hernia (11), anastomotic stenosis (2) and change in bowel movements (3). After colectomy, intrahospital complications were detected in *n* = 34 (26%) patients, mostly minor (*n* = 23) and complications after discharge in *n* = 28 patients (21.4%). In contrast, inhospital and out-of-hospital complications were detected in six (19.4%) and one (3.2%) patients after transanal surgery, respectively.

**Table 2:** Surgery indications and complications

		Number (%)
Indication for surgery (*n* = 162)	• Colonoscopy complications	4 (2.5%)
	• Resection of colorectal lesions	158 (97.5%)
Surgical approach (*n* = 162)	• Laparoscopy	84 (51.8%)
	• Reconverted laparoscopy	9 (5.5%)
	• Laparotomy	38 (23.1%)
	• Transanal surgery	31 (19.1%)
Type of surgery (*n* = 162)	• Expanded appendectomy	9 (5.6%)
	• Right hemicolectomy	74 (45.7%)
	• Left hemicolectomy	6 (3.7%)
	• Sigmoidectomy	15 (9.2%)
	• Rectum anterior resection	8 (4.9%)
	• Segmental resection	14 (8.6%)
	• Subtotal colectomy	5 (3.1%)
	• Transanal surgery	31 (19.1%)
Length of hospitalization (*n* = 162)		6 (IQR 4–9)
In hospital complications^a^ (*n* = 162)	• 0	122 (75.3%)
	• I	19 (11.7%)
	• II	9 (5.6%)
	• III	6 (3.7%)
	• IV	5 (3.1%)
	• V	1 (0.6%)
Complications after discharge (*n* = 160)		29 (18.1%)

^a^According to the Clavien-Dindo classification [19].

### Characteristics of the resected colorectal lesions

As we show in Table 3, most commonly surgically resected lesions were either right-sided (49.6%) or located in the rectum (22.2%). Median endoscopic size was 35 mm and most lesions were either sessile, flat or laterally spreading tumors. The lesions had a SMSA score above 12 in most cases (76.7%). An endoscopic resection was attempted in 23.5% of patients either in the work-up colonoscopy or in scheduled therapeutic colonoscopy. Median surgical size of the lesion was 25 mm and, as in the endoscopic histology, the most common histology was adenomatous (81.8%).

**Table 3:** Characteristics of colonic lesions resected surgically

		Number (%)
Location (*n* = 158)	• Appendix	10 (6.3%)
	• Cecum	28 (17.7%)
	• Ascending colon	26 (16.5%)
	• Hepatic flexure	14 (8.9%)
	• Transverse colon	13 (8.2%)
	• Splenic flexure	3 (1.9%)
	• Descending colon	9 (5.7%)
	• Sigmoid colon	20 (12.7%)
	• Rectum	35 (22.2%)
Endoscopic resection attempt (*n* = 158)	• Surgery directly	109 (68.9%)
	• In baseline colonoscopy	29 (18.4%)
	• In therapeutic colonoscopy	20 (12.7%)
Type of endoscopic resection (*n* = 153)	• No resection	118 (74.7%)
	• Incomplete resection	32 (20.3%)
	• Piecemeal resection	6 (3.8%)
	• En bloc resection	2 (1.3%)
Endoscopic size (mm) (*n* = 154)		35 (25–50)
Morphology (*n* = 155)	• Pedunculated	11 (7.0%)
	• Sessile	70 (44.3%)
	• Flat	17 (10.8%)
	• Flat-depressed	6 (3.8%)
	• Laterally spreading tumor	51 (32.3%)
Endoscopic histology (*n* = 158)	• Tubular adenoma with LGD	26 (16.4%)
	• Villous adenoma with LGD	51 (32.2%)
	• Adenoma with HGD	42 (26.5%)
	• Adenoma with intramucosal carcinoma	6 (3.8%)
	• Serrated lesion	7 (4.4%)
	• Serrated lesion with HGD	1 (0.6%)
	• Other histology	1 (0.6%)
	• No biopsy	24 (15.2%)
SMSA classification (*n* = 150)	• 6–8	4 (2.7%)
	• 9–12	31 (20.7%)
	• > 12	115 (76.7%)
Surgical size (mm) (*n* = 136)		25 (20–40)
Surgical histology (*n* = 158)	• No residual lesion	10 (6.3%)
	• Tubular adenoma with LGD	19 (12.0%)
	• Villous adenoma with LGD	45 (28.5%)
	• Adenoma with HGD	53 (33.5%)
	• Adenoma with intramucosal carcinoma	12 (7.8%)
	• Serrated lesion	9 (5.7%)
	• Serrated lesion with HGD	4 (2.5%)
	• Other	6 (3.8%)

*HGD* High grade dysplasia, *LGD* Low grade dysplasia, *SMSA* Size, Morphology, Site and Access

### Factors associated with surgery

During bivariate analysis, several factors related to the patient (age), screening program (FIT result and number of baseline colonoscopies performed), characteristics of the lesions detected (number of polyps and adenomas, adenoma size and classification according to the European guidelines for CRC screening), endoscopist quality metrics (ADR and number of colonoscopies performed), and hospital complexity were significantly associated with the surgery rate as shown in Table 2. With respect to colectomy, we also identified several associated variables as shown in supplementary Table 1.

Finally, in the multivariable logistic regression analysis we identified several variables independently associated with the risk of surgery: age ≥ 60 years (OR = 1.57, 95% CI 1.11–2.23), female sex (OR = 2.10, 95% CI 1.52–2.91), the European guidelines classification (high risk OR = 67.94, 95% CI 24.87–185.59; intermediate risk OR = 5.63, 95% CI 1.89–16.80: low risk OR = 1.43; 95% CI 0.36–5.75), the endoscopist’s ADR (Q4 OR = 0.44, 95% CI 0.28–0.68; Q3 OR = 0.44, 95% CI 0.27–0.71; Q2 OR = 0.71, 95% CI 0.44–1.14) and a tertiary hospital (OR = 0.54; 95% CI 0.38–0.79) (Table 4). After excluding transanal surgeries, the same variables were independently related to risk of colectomy: age ≥ 60 years (OR = 1.93, 95% CI 1.30–2.89), female sex (OR = 2.21, 95% CI 1.54–3.16), the European guidelines classification (high risk OR = 53.21, 95% CI 19.36–146.18; intermediate risk OR = 5.33, 95% CI 1.77–16.03; low risk OR = 1.07; 95% CI 0.24–4.78), the endoscopist’s ADR (Q4 OR = 0.37, 95% CI 0.23–0.61; Q3 OR = 0.48, 95% CI 0.29–0.79; Q2 OR = 0.58, 95% CI 0.34–1.00) and a tertiary hospital (OR = 0.57; 95% CI 0.38–0.85).

**Table 4:** Factors independently associated with colorectal surgery and colectomy in the logistic regression analysis

		Colorectal surgery (*n* = 162) Odds Ratio (95% CI)^a^	Colectomy (*n* = 131) Odds Ratio (95% CI)^a^
Sex (*n* = 15,707)	• Male	1	1
	• Female	2.10 (1.52–2.91)	2.21 (1.54–3.16)
Age (*n* = 15,707)	• < 60 years	1	1
	• ≥60 years	1.57 (1.11–2.23)	1.93 (1.30–2.89)
European guidelines classification (*n* = 15,707)	• No adenoma	1	1
	• Low risk	1.43 (0.36–5.75)	1.07 (0.24–4.78)
	• Intermediate risk	5.63 (1.89–16.80)	5.33 (1.77–16.03)
	• High risk	67.94 (24.87–185.59)	53.21 (19.36–146.18)
Endoscopist’s ADR (*n* = 15,459)	• Q1 (≤60%)	1	1
	• Q2 (60–65.3%)	0.71 (0.44–1.14)	0.58 (0.34–1.00)
	• Q3 (65.3–70.8%)	0.44 (0.27–0.71)	0.48 (0.29–0.79)
	• Q4 (> 70.8%)	0.44 (0.28–0.68)	0.37 (0.23–0.61)
Complexity of hospital (*n* = 15,707)	• Second level	1	1
	• Third level	0.54 (0.38–0.79)	0.57 (0.38–0.85)

*ADR* Adenoma detection rate, *CI* Confidence interval

^a^Odds Ratio and 95% CI calculated in the multivariable logistic regression analysis (forward conditional)

## Discussion

Our study reports the surgery rate in patients with nonmalignant lesions detected within a mass CRC screening program and the factors related to it. Most surgeries are related to resection of colorectal lesions and, exceptionally, to endoscopic complications. This information is extremely relevant to measure overtreatment risks in this setting. Fortunately, the surgery rate is low and the associated risk of mortality is as expected: one related death in 15,000 subjects. However, we have determined that not only factors associated with the patient and endoscopic findings but also the endoscopist’s performance measured with the ADR and hospital level of complexity are independently associated with surgery rate.

Our study has several strengths. The first is related to its population-based perspective. We have collected data from the first round of the Galician CRC screening program. During this initial round, FIT was offered to 721,349 subjects aged 50–69 years, colonoscopies were performed in seven hospitals and quality indicators of seventy-one endoscopists were collected in a centralized database. This database enabled us to calculate surgery rate according to the different variables available. Thus, we could accurately determine the risk of overtreatment in a mass screening program and the side effects related to surgery. There is not much information available to compare our data. As an example, in a retrospective study performed within the scope of the national English Bowel Cancer Screening Program, surgery rate in large polyps (≥20 mm flat or sessile) attained 21.7% [22]. Our data are not comparable because the European guidelines high risk group includes adenomas of any morphology ≥20 mm in size and/or ≥ 5 adenomas. In fact, most surgically resected lesions in our study were either sessile, flat or laterally spreading tumors (93%) with a SMSA > 12 in most cases. A French study evaluated the frequency and risk factors for the surgical resection of non-malignant polyps detected in a FIT based mass CRC screening program [12]. In this study, the surgery rate in patients with any polyp detected was 4.1% and was related with factors related to the size, location, histology, endoscopy center and the endoscopist. In our study, the surgery rate in the patients with at least one adenoma was clearly lower.

We have detected an association between the ADR and the surgery rate independent from the endoscopic findings. ADR is the endoscopist’s main quality indicator and has been associated with the risk of interval CRC [23], CRC death [24], detection of serrated polyps [25] and the adenoma detection during surveillance [26]. Although ADR is considered a surrogate for meticulous inspection of the colorectal mucosa, correlation with other important outcomes has never been found. In our case, we hypothesize that our findings reflect an association between the assessment of the mucosa and the endoscopists’ resection skills. Out results confirm that endoscopists are a risk factor for surgery in patients with polyps detected in a screening program [12]. Nevertheless, we must draw attention to the high ADR of the endoscopists taking part in the Galician screening program. Although an ADR above 45% is recommended in a FIT-based screening programs [27], in our case 75% of endoscopists attained a 60% ADR.

Our study has several limitations related to quality of the data collected in the CRC screening database. First, we used the CMBD to identify all the colorectal surgeries. Although we do not have information regarding the accuracy of the data obtained from the Spanish CMBD, an evaluation of the ICD-9-CM for CRC in an Italian administrative database showed a sensitivity ranging between 98 and 99% [28]. Unfortunately, information regarding location, morphology of the most advanced lesion, SMSA classification or the visual predicted histology of the lesions detected was not stored. We cannot provide information regarding on the visual suspicion of malignancy of the polyp that could explain some of the referrals to surgery. Additionally this information could explain one of the most striking findings of our study. Although males have an increased risk of advanced neoplasia detection in CRC screening [29] and account for 75% of the high risk lesions detected [30], in our study, females have an increased risk of surgery. The reason is unclear and we suggest it may be related to differences in the natural history of CRC. There is evidence that the serrated carcinogenic pathway, through hypermethylation and BRAF V600E mutation, with flat or sessile serrated lesions located proximally [31, 32], is more common among females and this could explain our findings. In fact, as the study by Le Roy et al. [12] shows, location is a risk factor for surgery in polyps detected in a screening program. Unfortunately, this information was unavailable for patients not requiring surgery.

Colorectal complications and mortality in our study are within the ranges expected. Data analyzed from a National Surgical Quality Improvement Program from 2011 to 2014, including 12,732 patients who underwent elective surgery for nonmalignant colorectal lesions, revealed a 0.7% 30-day mortality rate and 14% risk of major postoperative adverse events [10]. We also analyzed the long term complications that mainly impair the subject’s quality of life [33]. In contrast, endoscopic resection is more cost-effective, has few side effects, complications no greater than 1 to 2% and mortality below 1/10,000 [2]. In large colorectal lesions, endoscopic resection-related mortality ranges between 0 and 0.08% [34, 35]. Despite professional society guidelines and recommendations [9, 36], colectomies for benign colon lesions have increased in the last few years. In the US, surgery incidence for nonmalignant lesions has increased from 5.9/100,000 to 9.4/100,000 adults in 2000 and 2014, respectively [10].

Our study highlights the need for improved endoscopic resection techniques. First, endoscopists need to be trained specifically in visual assessment of colorectal lesions and in resection techniques and their results should be continuously monitored. In this sense, we require contrasted quality indicators adapted to each screening scenario (FIT, colonoscopy). Quality indicators such as visual diagnostic yield, complete resection, complications, relapse and colectomy rates in large colorectal polyps should be monitored both per endoscopist and per endoscopy unit. However, complex endoscopic resection techniques such as submucosal dissection and endoscopic full thickness resection should be available and patients should be referred to centralized units where these techniques are performed on a regular basis [36].

## Conclusions

To conclude, the surgery rate in patients with nonmalignant lesions detected in a mass screening program is low and mainly associated with treatment of unresectable polyps. Although complications related to surgery are acceptable, this is an area both endoscopists and endoscopy units can improve upon. In order to reduce the number of subjects referred to surgery, we need to improve the endoscopist resection skills and centralized units for complex techniques should be available. Finally, we require endoscopic resection quality indicators that enable us to continuously monitor endoscopic resection results.

## Supplementary Information

**Additional file 1: Supplementary Table 1.** Baseline characteristics and colectomy rate according to dependent variables.

## Data Availability

The datasets used and/or analysed during the current study are available from the corresponding author on reasonable request.

## Abbreviations

ADR: Adenoma detection rate
AJCC: American Joint Committee on Cancer
ASA: American Society of Anesthesiologists’ Physical Status
CI: Confidence interval
CRC: Colorectal cancer
FIT: Fecal immunochemical test
Hb: Hemoglobin
HGD: High grade dysplasia
IQR: Interquartile range
LGD: Low grade dysplasia
OR: Odds ratio
PS: Performance status
SMSA: Size, Morphology, Site and Access

## References

[1] Cancer Today. International Agency for Research on Cancer [https://gco.iarc.fr/today/home]. Accessed 21 July 2021.

[2] Cubiella J, Marzo-Castillejo M, Mascort-Roca JJ, Amador-Romero FJ, Bellas-Beceiro B, Clofent-Vilaplana J, Carballal S, Ferrándiz-Santos J, Gimeno-García AZ, Jover R, Mangas-Sanjuán C, Moreira L, Pellisè M, Quintero E, Rodríguez-Camacho E, Vega-Villaamil P, Sociedad Española de Medicina de Familia y Comunitaria y Asociación Española de Gastroenterología (2018). Clinical practice guideline. Diagnosis and prevention of colorectal cancer. 2018 Update. Gastroenterol Hepatol.

[3] Zorzi M, Fedeli U, Schievano E, Bovo E, Guzzinati S, Baracco S, Fedato C, Saugo M, Dei Tos AP (2015). Impact on colorectal cancer mortality of screening programmes based on the faecal immunochemical test. Gut.

[4] Levin TR, Corley DA, Jensen CD, Schottinger JE, Quinn VP, Zauber AG, Lee JK, Zhao WK, Udaltsova N, Ghai NR, Lee AT, Quesenberry CP, Fireman BH, Doubeni CA (2018). Effects of Organized Colorectal Cancer Screening on Cancer Incidence and Mortality in a Large Community-Based Population. Gastroenterology.

[5] Criteria for appraising the viability, effectiveness and appropriateness of a screening programme Updated 23 October 2015 [https://www.gov.uk/government/publications/evidence-review-criteria-national-screening-programmes/criteria-for-appraising-the-viability-effectiveness-and-appropriateness-of-a-screening-programme]. Accessed 21 July 2021.

[6] Vermeer NCA, Snijders HS, Holman FA, Liefers GJ, Bastiaannet E, van de Velde CJH, Peeters KCMJ (2017). Colorectal cancer screening: systematic review of screen-related morbidity and mortality. Cancer Treat Rev.

[7] Kalager M, Wieszczy P, Lansdorp-Vogelaar I, Corley DA, Bretthauer M, Kaminski MF (2018). Overdiagnosis in colorectal Cancer screening: time to acknowledge a blind spot. Gastroenterology.

[8] Zauber AG, Winawer SJ, O’Brien MJ, Lansdorp-Vogelaar I, van Ballegooijen M, Hankey BF, Shi W, Bond JH, Schapiro M, Panish JF, Stewart ET, Waye JD (2012). Colonoscopic polypectomy and long-term prevention of colorectal-cancer deaths. N Engl J Med.

[9] Ferlitsch M, Moss A, Hassan C, Bhandari P, Dumonceau J-M, Paspatis G, Jover R, Langner C, Bronzwaer M, Nalankilli K, Fockens P, Hazzan R, Gralnek IM, Gschwantler M, Waldmann E, Jeschek P, Penz D, Heresbach D, Moons L, Lemmers A, Paraskeva K, Pohl J, Ponchon T, Regula J, Repici A, Rutter MD, Burgess NG, Bourke MJ (2017). Colorectal polypectomy and endoscopic mucosal resection (EMR): European Society of Gastrointestinal Endoscopy (ESGE) clinical guideline. Endoscopy.

[10] Peery AF, Cools KS, Strassle PD, McGill SK, Crockett SD, Barker A, Koruda M, Grimm IS (2018). Increasing Rates of Surgery for Patients With Nonmalignant Colorectal Polyps in the United States. Gastroenterology.

[11] Ma C, Teriaky A, Sheh S, Forbes N, Heitman SJ, Jue TL, Munroe CA, Jairath V, Corley DA, Lee JK (2019). Morbidity and mortality after surgery for nonmalignant colorectal polyps: a 10-year Nationwide analysis. Am J Gastroenterol.

[12] Le Roy F, Manfredi S, Hamonic S, Piette C, Bouguen G, Riou F, Bretagne J-F (2016). Frequency of and risk factors for the surgical resection of nonmalignant colorectal polyps: a population-based study. Endoscopy.

[13] Cubiella J, González A, Almazán R, Rodríguez-Camacho E, Fontenla Rodiles J, Domínguez Ferreiro C, et al. pT1 colorectal Cancer detected in a colorectal Cancer mass screening program: treatment and factors associated with residual and Extraluminal disease. Cancers. 12(9):2530. 10.3390/cancers12092530. 10.3390/cancers12092530 PMC7565413 32899974

[14] von Karsa L, Patnick J, Segnan N, Atkin W, Halloran S, Lansdorp-Vogelaar I, Malila N, Minozzi S, Moss S, Quirke P, Steele RJ, Vieth M, Aabakken L, Altenhofen L, Ancelle-Park R, Antoljak N, Anttila A, Armaroli P, Arrossi S, Austoker J, Banzi R, Bellisario C, Blom J, Brenner H, Bretthauer M, Camargo Cancela M, Costamagna G, Cuzick J, Dai M, European Colorectal Cancer Screening Guidelines Working G (2013). European guidelines for quality assurance in colorectal cancer screening and diagnosis: overview and introduction to the full supplement publication. Endoscopy.

[15] Edge SB, Byrd DR, Compton CC, Fritz AG, Greene FL, Trotti A. AJCC Cancer Staging Manual. 7th ed. New York: Springer-Verlag; 2010.

[16] Jover R, Herráiz M, Alarcón O, Brullet E, Bujanda L, Bustamante M, Campo R, Carreño R, Castells A, Cubiella J, García-Iglesias P, Hervás AJ, Menchén P, Ono A, Panadés A, Parra-Blanco A, Pellisé M, Ponce M, Quintero E, Reñé JM, Sánchez del Río A, Seoane A, Serradesanferm A, Soriano Izquierdo A, Vázquez Sequeiros E, Spanish Society of Gastroenterology, Spanish Society of Gastrointestinal Endoscopy Working Group (2012). Clinical practice guidelines: quality of colonoscopy in colorectal cancer screening. Endoscopy.

[17] Atkin WS, Valori R, Kuipers EJ, Hoff G, Senore C, Segnan N, Jover R, Schmiegel W, Lambert R, Pox C (2012). European guidelines for quality assurance in colorectal cancer screening and diagnosis. First Edition--Colonoscopic surveillance following adenoma removal. Endoscopy.

[18] Ministerio de Sanidad, Servicios Sociales e Igualdad [internet]. Hospital discharge records in the national health system. CMBD. [http://www.msssi.gob.es/en/estadEstudios/estadisticas/cmbdhome.htm]. Accessed 21 July 2021.

[19] Clavien PA, Barkun J, De Oliveira ML, Vauthey JN, Dindo D, Schulick RD, De Santibañes E, Pekolj J, Slankamenac K, Bassi C, Graf R, Vonlanthen R, Padbury R, Cameron JL, Makuuchi M (2009). The clavien-dindo classification of surgical complications: five-year experience. Ann Surg.

[20] Lambert R, Lightdale CJ (2003). The Paris endoscopic classification of superficial neoplastic lesions: Esophagus, stomach, and colon - Paris, France November 30 to December 1, 2002. Gastrointest Endosc.

[21] Sidhu M, Tate DJ, Desomer L, Brown G, Hourigan LF, Lee EYT, Moss A, Raftopoulos S, Singh R, Williams SJ, Zanati S, Burgess N, Bourke MJ (2018). The size, morphology, site, and access score predicts critical outcomes of endoscopic mucosal resection in the colon. Endoscopy.

[22] Lee TJW, Rees CJ, Nickerson C, Stebbing J, Abercrombie JF, McNally RJQ, Rutter MD (2013). Management of complex colonic polyps in the English bowel Cancer screening Programme. Br J Surg.

[23] Kaminski MF, Regula J, Kraszewska E, Polkowski M, Wojciechowska U, Didkowska J, Zwierko M, Rupinski M, Nowacki MP, Butruk E (2010). Quality indicators for colonoscopy and the risk of interval cancer. N Engl J Med.

[24] Corley DA, Jensen CD, Marks AR, Zhao WK, Lee JK, Doubeni CA, Zauber AG, de Boer J, Fireman BH, Schottinger JE, Quinn VP, Ghai NR, Levin TR, Quesenberry CP (2014). Adenoma detection rate and risk of colorectal cancer and death. N Engl J Med.

[25] Zorzi M, Senore C, Da Re F, Barca A, Bonelli LA, Cannizzaro R, de Pretis G, Di Furia L, Di Giulio E, Mantellini P, Naldoni C, Sassatelli R, Rex DK, Zappa M, Hassan C, Equipe Working Group (2017). Detection rate and predictive factors of sessile serrated polyps in an organised colorectal cancer screening programme with immunochemical faecal occult blood test: the EQuIPE study (evaluating quality indicators of the performance of endoscopy). Gut.

[26] Mangas-Sanjuan C, Zapater P, Cubiella J, Murcia Ó, Bujanda L, Hernández V, Martínez-Ares D, Pellisé M, Seoane A, Lanas Á, Nicolás-Pérez D, Herreros-de-Tejada A, Chaparro M, Cacho G, Fernández-Díez S, Marín-Gabriel J-C, Quintero E, Castells A, Jover R, COLONPREV study investigators (2018). Importance of endoscopist quality metrics for findings at surveillance colonoscopy: the detection-surveillance paradox. United Eur Gastroenterol J.

[27] Cubiella J, Castells A, Andreu M, Bujanda L, Carballo F, Jover R, Lanas Á, Morillas JD, Salas D, Quintero E, COLONPREV study investigators (2017). Correlation between adenoma detection rate in colonoscopy- and fecal immunochemical testing-based colorectal cancer screening programs. United Eur Gastroenterol J.

[28] Cozzolino F, Bidoli E, Abraha I, Fusco M, Giovannini G, Casucci P, Orso M, Granata A, De Giorgi M, Collarile P, Ciullo V, Vitale MF, Cirocchi R, Orlandi W, Serraino D, Montedori A, D.I.V.O. Group (2018). Accuracy of colorectal cancer ICD-9-CM codes in Italian administrative healthcare databases: a cross-sectional diagnostic study. BMJ Open.

[29] Cubiella J, Castro I, Hernandez V, González-Mao C, Rivera C, Iglesias F, Alves MT, Cid L, Soto S, De-Castro L, Vega P, Hermo JA, Macenlle R, Martínez A, Estevez P, Cid E, Herreros-Villanueva M, Portillo I, Bujanda L, Fernández-Seara J, COLONPREV study investigators (2014). Diagnostic accuracy of fecal immunochemical test in average- and familial-risk colorectal cancer screening. United Eur Gastroenterol J.

[30] Cubiella J, Carballo F, Portillo I, Cruzado Quevedo J, Salas D, Binefa G, Milà N, Hernández C, Andreu M, Terán Á, Arana-Arri E, Ono A, Valverde MJ, Bujanda L, Hernández V, Morillas JD, Jover R, Castells A (2016). Incidence of advanced neoplasia during surveillance in high- and intermediate-risk groups of the European colorectal cancer screening guidelines. Endoscopy.

[31] Chacko L, Macaron C, Burke CA (2015). Colorectal Cancer screening and prevention in women. Dig Dis Sci.

[32] McCashland TM, Brand R, Lyden E, Garmo P (2001). Gender differences in colorectal polyps and tumors. Am J Gastroenterol.

[33] Giglia MD, Stein SL (2019). Overlooked long-term complications of colorectal surgery. Clin Colon Rectal Surg.

[34] Ahlenstiel G, Hourigan LF, Brown G, Zanati S, Williams SJ, Singh R, Moss A, Sonson R, Bourke MJ, Australian colonic endoscopic mucosal resection (ACE) study group (2014). Actual endoscopic versus predicted surgical mortality for treatment of advanced mucosal neoplasia of the colon. Gastrointest Endosc.

[35] Hassan C, Repici A, Sharma P, Correale L, Zullo A, Bretthauer M, Senore C, Spada C, Bellisario C, Bhandari P, Rex DK (2016). Efficacy and safety of endoscopic resection of large colorectal polyps: a systematic review and meta-analysis. Gut.

[36] Kaltenbach T, Anderson JC, Burke CA, Dominitz JA, Gupta S, Lieberman D, Robertson DJ, Shaukat A, Syngal S, Rex DK (2020). Endoscopic removal of colorectal lesions—recommendations by the US multi-society task force on colorectal Cancer. Gastroenterology.

