# Supplementary material for: Overtreatment in nonmalignant lesions detected in a colorectal cancer screening program: a retrospective cohort study
Source: BMC Cancer. 2021 Jul 29;21:869. doi: 10.1186/s12885-021-08606-w (PMC8323280; doi:10.1186/s12885-021-08606-w)
Supplement: Supplementary file 1 — Additional file 1: Supplementary Table 1. Baseline characteristics and colectomy rate according to dependent variables. [file 12885_2021_8606_MOESM1_ESM.docx]

**Supplementary Table 1.** Baseline characteristics and colectomy rate according to dependent variables.

|  | | Patients not requiring colectomy (n=15,576) | Colectomy  (n=131) | Colectomy rate % (95% CI) | Significance^1^ |
| --- | --- | --- | --- | --- | --- |
| Sex  (n=15,707) | - Female - Male | 6,720 (43.1%)  8,856 (56.9%) | 60 (45.8%)  71 (54.2%) | 0.88 (0.66-1.11)  0.79 (0.61-1.98) | 0.5 |
| Age  (n=15,707) | - <60 years - ≥60 years | 6,869 (44.1%)  8,707 (55.9%) | 34 (26.0%)  97 (74.0%) | 0.49 (0.33-0.66)  1.10 (0.88-1.32) | <0.001 |
| PS  (n=15, 383) | - 0 - 1 | 12,410 (81.3%)  2,844 (18.6%) | 110 (84.0%)  19 (16.0%) | 0.88 (0.71-1.04)  0.66 (0.37-0.96) | 0.4 |
| ASA  (n=15,383) | - I - II - III | 8,894 (58.4%)  5,400 (35.5%)  960 (6.1%) | 78 (60.5%)  44 (34.1%)  7 (5.4%) | 0.87 (0.68-1.06)  0.81 (0.57-1.05)  0.72 (0.19-1.26) | 0.9 |
| Fecal Hb (n=15,707) | - <100 µg/g - 100-200 µg/g - >200 µg/g | 10,403 (66.8%)  2,945 (18.9%)  2,228 (14.3%) | 64 (48.9%)  31 (23.7%)  36 (27.5%) | 0.61 (0.46-0.76)  1.08 (0.70-1.45)  1.59(1.07-2.10) | <0.001 |
| N.colonoscopies  (n=15,707) | - One - At least two | 13,583 (87.2%)  1,993 (12.8%) | 96 (73.3%)  35 (26.7%) | 0.89 (0.73-1.05)  1.97 (1.37-2.58) | <0.001 |
| Number of polyps (n=15,707) | | 2 (1-4) | 3 (1-5) |  | <0.001 |
| Number of adenomas (n=15,707) | | 1 (0-3) | 2 (1-4) |  | <0.001 |
| Adenoma size (mm) (n=15,707) | | 5 (0-12) | 20 (7-34) |  | <0.001 |
| European guidelines classification  (n=15,707) | - No adenoma - Low risk - Intermediate risk - High risk | 4,650 (29.9%)  3,627 (23.3%)  4,180 (26.8%)  3,119 (20.0%) | 4 (3.1%)  3 (2.3%)  16 (12.2%)  108 (82.4%) | 0.08 (0.00-0.17)  0.08 (-0.01-0.18)  0.38 (0.19-0.57)  3.35 (2.73-3.97) | <0.001 |
| Endoscopist’s ADR  (n=15,459) | - Q1 (≤60%) - Q2 (60-65.3%) - Q3 (65.3-70.8%) - Q4 (>70.8%) | 2,659 (17.3%)  4,000 (26.1%)  4,183 (27.3%)  4,486 (29.3%) | 36 (27.7%)  29 (22.3%)  32 (24.6%)  33 (25.4%) | 1.33 (0.90-1.76)  0.72 (0.46-0.98)  0.76 (0.50-1.02)  0.73 (0.48-0.98) | 0.02 |
| Endoscopist’s number of colonoscopies  (n=15,459) | - Q1 (≤57) - Q2 (58-278) - Q3 (279-507) - Q4 (>507) | 198 (1.3%)  1,652 (10.8%)  5,344 (34.9%)  8,135 (53.1%) | 2 (1.5%)  13 (10.0%)  35 (26.9%)  80 (61.5%) | 1.00 (-0.38-2.38)  0.78 (0.36-1.20)  0.56 (0.43-0.65)  0.97 (0.76-1.19) | 0.2 |
| Complexity of the hospital (n=15,707) | - Third level - Second level | 7,160 (46.0%)  8,416 (54.0%) | 42 (32.1%)  89 (67.9%) | 0.58 (0.41-0.76)  1.04 (0.83-1.26) | 0.002 |

1Significance in the univariant analysis using the Chi-square test for qualitative variables and the Student’s t test for cuantitative variables.

ADR, adenoma detection rate; ASA, American Society of Anesthesiologists’ Physical Status; CI, confidence interval; Hb, hemoglobin; IQR, interquartile range; PS, performance status.
